# Molecular Pathogenesis of Intrahepatic Cholestasis of Pregnancy

**DOI:** 10.1155/2021/6679322

**Published:** 2021-05-30

**Authors:** Jianping Xiao, Zeying Li, Yutong Song, Yujie Sun, Hanfei Shi, Daozhen Chen, Yan Zhang

**Affiliations:** ^1^Department of Gynecology and Obstetrics, Wuxi Maternal and Child Health Hospital, the Affiliated Hospital of Nanjing Medical University, Wuxi 214002, China; ^2^The First Clinical Medical College, Nanjing Medical University, Nanjing 211166, China

## Abstract

Intrahepatic cholestasis of pregnancy (ICP) is a pregnancy-specific liver disease. The maternal symptoms are characterized by skin pruritus and elevated bile acids, causing several adverse outcomes for fetuses, including an increased risk of preterm birth, meconium-stained amniotic fluid, neonatal depression, respiratory distress syndrome, and stillbirth. Genetic, hormonal, immunological, and environmental factors contribute to the pathogenesis of ICP, and the estrogen-bile acid axis is thought to play a dominant role. The advances in the past 10 years uncover more details of this axis. Moreover, dysregulation of extracellular matrix and oxygen supply, organelle dysfunction, and epigenetic changes are also found to cause ICP, illuminating more potential drug targets for interfering with. Here, we summarize the molecular pathogenesis of ICP with an emphasis on the advancement in the past 10 years, aiming to give an updated full view of this field.

## 1. Introduction

Intrahepatic cholestasis of pregnancy (ICP) is a pregnancy-specific liver disease characterized by skin pruritus and elevated bile acids [1]. The maternal symptoms usually appear in the second or third trimester of pregnancy and rapidly resolve after delivery. Under rare circumstances, ICP may also present as early as the first trimester [2]. There is a lack of consensus in the diagnostic criteria for ICP. Most guidelines agree on the requirement of pruritus and abnormal liver function. The American College of Gastroenterologists (ACG), the European Association for the Study of the Liver (EASL), and the Society for Maternal-Fetal Medicine (SMFM) only mention persistent pruritus that resolves with delivery and bile acid concentrations >10 *μ*mol/L for diagnosis. South Australia Maternal and Neonatal Community of Practice (SAMNCP) notes that while bile acid concentrations >10 *μ*mol/L are suggestive, values > 15 *μ*mol/L are diagnostic [3, 4]. For fetuses, ICP causes more adverse outcomes than mothers, including an increased risk of preterm birth, meconium-stained amniotic fluid, neonatal depression, respiratory distress syndrome, and stillbirth [5]. Generally, earlier occurrence of ICP is associated with more serious adverse prenatal outcomes. Ursodeoxycholic acid (UDCA), which can relieve pruritus and decrease bile acid level, is the most common obstetric prescription medicine for ICP, although its beneficial effect on fetal outcomes is controversial [1].

Bile acids (BAs) are cholesterol-derived molecules produced by the liver that exert hormone-like metabolic effects by binding to the nuclear farnesoid *X* receptors (FXRs) and the membrane-bound Takeda *G* protein-coupled receptors 5 (TGR5) [6, 7]. Activated FXR can directly induce the expression of nuclear receptors that is responsible for the transport of bile acids from the lumen to hepatocytes and at the same time induces the expression of the main transporter of bile from the liver [8]. Unlike FXR, TGR5 is a plasma membrane-bound bile acid receptor that indirectly affects gene expression by initiating specific downstream signaling pathways [9]. Another main signal pathway is PPAR-*γ*/NF-*κ*b. Peroxisome proliferator-activated receptor-*γ* (PPAR-*γ*) can trigger antioxidant activity and inhibit stress response [10]. NF-*κ*B is a nuclear transcription factor, regulating the expression of a variety of cytokines [11], and placental tissue NF-*κ*B high expression levels play a role in the pathogenesis of ICP. PPAR can reduce the concentration of reactive oxygen species (ROS), indirectly acting on NF-*κ*B [12].

The pathogenesis of ICP is multifactorial, with an interaction of genetic, hormonal, immunological, and environmental factors [13]. Although the exact molecular mechanism of how ICP occurs is still elusive, the advances in the past 10 years uncover more details. The estrogen-bile acid axis still accounts for the majority of research studies, with more associated genes and signaling pathways found. Moreover, dysregulation of extracellular matrix and oxygen supply, organelle dysfunction, epigenetic changes, and so on are found to cause ICP.

Here, we summarize the recent advances in our understanding of how ICP occurs. The conclusions of earlier research studies were also briefly mentioned to depict an overall view of the pathogenesis of ICP.

## 2. The Role of Genetic Factors and Other Epigenetic Regulators

Among the genetic factors, mutations of ABCB4 (MDR3) involve in the biliary secretion of phospholipids, which can be observed in approximately 16% of all ICP cases. Biliary phospholipids are responsible for neutralization of the detergent effects of hydrophobic bile salts by the formation of mixed micelles. Thus, ABCB4 protein defects can lead to intrahepatic cholestasis. However, ABCB4 protein-coding disorders can also occur in hereditary low phospholipid-associated cholelithiasis (LPAC) and drug-induced cholestasis [14]. Moreover, the mutations of ABCB11 (BSEP) protein-encoding gene may cause ICP, and several studies found ABCB11 mutations in women affected with ICP [15, 16]. According to the OMIM database, there is no documented evidence regarding the association between ABCB11 gene and ICP, and this allele is relatively common in the South Asian population. Thus, it seems not likely to be a pathogenic variant and needs further case reports [17]. In recent years, many studies also addressed the role of the ATP8B 1 and TJP2 genes in ICP susceptibility and identified some possible effects loci associated with ICP [18]. Jeremy et al. also found that a total of 8 mutations in the ANO8 gene were identified in eight of the 151 individuals, which may provide new insights into the genetic architecture of ICP disease [19].

**Figure 1:**
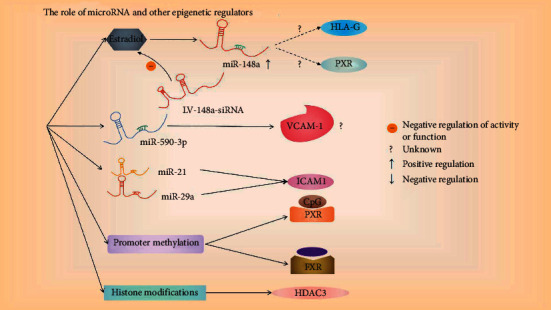
The role of genetic regulatory factors in the pathogenesis of ICP. Estradiol (inhibited by LV-148a-siRNA) can upregulate miR-148a, and its potential targets are HLA-G and PXR; miR-590-3p can regulate the expression of VCAM-1; miR-21 and miR-29a can inhibit ICAM1. In addition, the CpG sites of FXR and PXR promoters are less methylated in ICP patients. For histone modification, the expression of HDAC3 in ICP is low. The question mark indicates that it has not been confirmed, the dotted line indicates the potential target, and the specific regulation path is shown in the arrow in the figure.

The significance of epigenetic regulation (microRNAs, DNA methylation, and histone modification) on gene expression is gradually appreciated in ICP (Figure 1). MicroRNAs are a class of small noncoding RNA molecules (containing about 22 nucleotides) found in plants, animals, and some viruses that function in RNA silencing and posttranscriptional regulation of gene expression [20]. In ICP patients, miR-148a levels were markedly upregulated in the placenta and peripheral blood [21]. Rao et al. further showed that estradiol significantly upregulated miR-148a expression and LV-148a-siRNA inhibits the function of estradiol on total bile acid secretion, suggesting that miR-148a mediates partial function of estrogen. It also seems that HLA-G and pregnane *X* receptor (PXR) are potential targets of miR-148a, which needs further experimental confirmation [22]. In recent experiments by Jing et al., it was found that miR-148b-3p may negatively regulate the expression of GLUT1 in placental trophoblast cells to participate in the glucose metabolism of the offspring of maternal cholestasis [23]. Another microRNA, miR-590-3p, directly regulates vascular cell adhesion protein 1 (VCAM-1) expression, which is inhibited by taurocholic acid and decreases in ICP patients, although other studies showed opposite results [24, 25]. Whether VCAM-1 increases or decreases in ICP patients, its expression level dynamics need systematic exploration. In addition, Jiang et al. confirmed that miR-21 and miR-29a can directly target and inhibit the expression of ICAM1 [26].

Promoter methylation is usually associated with gene silencing. Cabrerizo et al. showed that the CpG sites of the distal and proximal FXR promoter and distal PXR promoter are less methylated in ICP patients [27]. Moreover, Yi et al. showed that a circulating cell-free fetal DNA, RAS-association domain family 1, isoform A (RASSF1A), is hypermethylated, which may be used as a diagnostic marker for ICP [28]. For histone modifications, Shao et al. showed lower expression of HDAC3 in ICP [29]. Whether HDAC3 or other HDACs affect the expression of inflammatory cytokines (IL-18, IL-12 and TNF-*α*) needs further study.

## 3. The Role of Estrogen and Other Hormones

**Figure 2:**
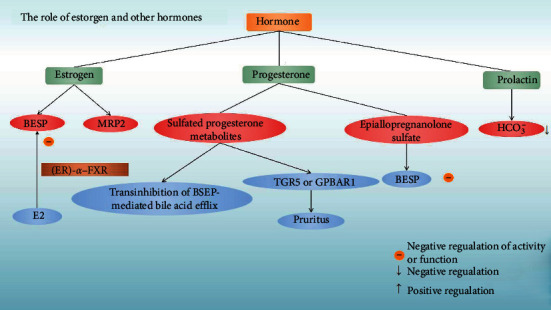
The role of hormones in the pathogenesis of ICP. Estrogen can regulate two proteins related to bile acid homeostasis, BESP, and MRP2, among which E2 can inhibit BESP through the interaction of ER-*α* and FXR; the metabolism of sulfated progesterone can activate GPBAR1 or TGR5 and cause transinhibition of BSEP-mediated bile acid efflux. Epiallopregnanolone sulfate can inhibit the expression of BESP. Prolactin can lead to a decrease in the concentration of bicarbonate in the blood. Adjustment events are displayed with arrows.

The link between estrogen and ICP is obvious (Figure 2). ICP mainly occurs during the last trimester, and twin pregnancy also has a higher risk of ICP. The association of ICP with the third trimester has led many researchers to study changes in steroidogenesis in women with ICP, with findings of lower levels of estrogen and dehydroepiandrosterone sulfate [30]. Most research studies showed that there are higher metabolites of progesterone in patients with ICP, with the main changes occurring in their sulfates [31, 32].

The bile salt export pump (BSEP) is responsible for the secretion of bile acids. Estrogen was reported to decrease the protein level of bile salt export pump (BSEP) and multidrug resistant-associated protein 2 (MRP2), both of which are involved in bile acid homeostasis [13]. In animal models, Song et al. showed that BSEP transcription was markedly repressed in the later stages of pregnancy and immediately recovered after parturition. They also found the inverse correlation between the transcriptional dynamics of BSEP and serum estrogen 17*β*-estradiol (E2) levels, and E2 repressed BSEP expression in human primary hepatocytes, Huh 7 cells [33].

Recently, Chen et al. illuminated how E2 transcriptionally repressed BSEP through an interaction between estrogen receptor- (ER-) *α* and farnesoid *X* receptor (FXR) [34]. Through quantitative chromatin immunoprecipitation assays, Chen et al. found that coactivator peroxisome proliferator-activated receptor-*γ* coactivator-1 was predominantly recruited to the BSEP promoter upon FXR activation, and its recruitment was decreased by E2 treatment, while recruitment of nuclear receptor corepressor was markedly increased upon E2 treatment. They also showed that domains AD and CF in ER-*α* mediated ligand-independent and ligand-dependent transrepression on BSEP, respectively, through interacting with FXR.

Placental P-glycoprotein (P-gp) plays a significant role in controlling transplacental digoxin transfer rate. Wang et al. showed that E2 could upregulate P-gp expression and reduce transplacental digoxin transfer rate in mice, suggesting the necessity of individualized transplacental digoxin treatment for fetal heart failure, especially for ICP pregnancy [35]. Obeticholic acid (OCA) can prevent cholestasis in pregnant mice caused by E2-caused intrauterine growth restriction (IUGR) by inhibiting placental oxidative stress and maintaining bile acid homeostasis [36].

Compared with estrogen, the role of progesterone in ICP pathogenesis is less studied. Administration of progesterone to rodents has little effect on bile secretion, while sulfated progesterone metabolites induce transinhibition of BSEP-mediated bile acid efflux [13]. Abu-Hayyeh et al. further showed that epiallopregnanolone sulfate (a 3*β*-sulfated progesterone metabolite) was raised in the serum of ICP patients, and epiallopregnanolone sulfate inhibited FXR-mediated BSEP expression and function through competitively inhibiting ligand-dependent FXR activation [37]. The study also found that a progesterone metabolite, epipentaerythritol ketone sulfate (PM5 S) in the serum of patients with ICP, was physiologically elevated, indicating that it may be one of the causes of ICP [38]. Besides involvement of bile acid regulation, sulfated progesterone metabolites were also linked to the pruritus symptom of ICP. Glantz et al. found that amelioration of pruritus by UDCA is associated with decreased progesterone disulphates in urine [39]. Moreover, sulfated progesterone metabolites were found to activate *G* protein-coupled bile acid receptor 1 (GPBAR1 or TGR5), which mediates the pruritus reaction, suggesting a potential therapeutic target for itch management in ICP [40].

Last, Bulaeva et al. showed that in ICP rat models, the main effect of prolactin was stimulation of bicarbonate clearance and inhibition of reabsorption, which led to a decrease in bicarbonate blood concentration [41]. How this effect of prolactin contributes to the pathogenesis of ICP still needs more clinical research studies.

## 4. The Role of Hypoxia

Hypoxia is a classic characteristic of the molecular microenvironment in multiple disease progression [42]. ICP is complicated by acute placental-fetal hypoxia, and direct molecular evidence for this is acuminating during the past 10 years.

Corticotropin-releasing hormone (CRH) and urocortin (UCN) are vasodilatory regulators of blood flow in the placenta. Downregulated UCN expression in ICP has been reported by several groups [43–45]. The study by Xu et al. also showed that the transcription and translation of UCN changed during the process of ICP in early pregnancy [46]. Moreover, He et al. showed that compared with normal pregnancy, the ICP patients had 29% reduction in the volume of placental lobular villi vessels, which is mainly a result of reduction of lobular villi vessels with smooth muscles [47].

Unlike CRH/UCN, the level of hypoxia-inducible factor 1-alpha (HIF-1*α*), HIF-2*α*, and protein regulated in development and DNA damage response 1 (REDD1), which play a significant role in the reaction to hypoxia and oxidative stress and regulate glucose metabolism, increases in the placental tissues of ICP patients, and this increase is mainly at the posttranscriptional level [48, 49]. Increased HIF-1*α* was reported to increase P53 level, and the unbalance of HIF-1*α* and P53 may cause fetal hypoxia and stillbirth [50]. Short-term hypoxia can also induce upregulation of VEGF expression in ICP placenta, and this adaptive change is probably a protective mechanism of fetus in ICP [51].

## 5. The Role of the Immunological System

**Figure 3:**
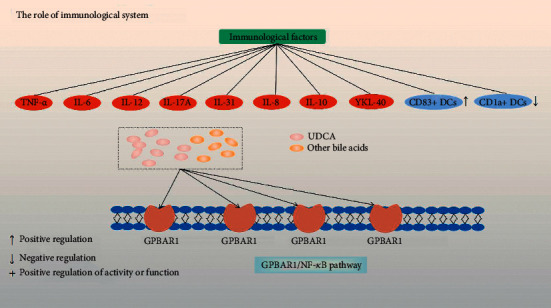
The role of inflammatory factors in the pathogenesis of ICP. The discovered ones include TNF-*α*, IL-6, IL-12, IL-17A, IL-31, IL-8, IL-10, and YKL-40, which are represented by red circles. In ICP patients, the number of DCs in the immune cell subgroup CD83+ is higher, while the number of DCs in CD1a+ is lower, indicated by blue circles. In addition, there is the GPBAR1/NF-*κ*B pathway, and UCDA can compete with other bile acids for the GPBAR1 receptor. Regulation events are displayed with arrows.

The immunological basis in the pathogenesis of ICP was summarized well by Larson et al. [52]. Here, we only mentioned several newest advances in this area after 2016 and also presented as a schematic diagram (Figure 3).

Besides tumor necrosis factor-alpha (TNF-*α*), interleukin 6 (IL-6), IL-12, and IL-17A, Basile et al. showed that IL-31, which is involved in the pathogenesis of pruritic inflammatory skin diseases, increases in ICP patients [53]. In addition, studies also confirmed the role of IL-8 and IL-10 in intrahepatic cholestasis of pregnancy and the level of YKL-40 increased [54, 55]. For immune cell subpopulation distribution, Kong et al. showed that there were more CD83+ dendritic cells (DCs) in the placenta from women with ICP than in normal pregnancies, while the number of decidual CD1a + DCs was significantly lower in ICP than in normal pregnant women [56]. Moreover, Zhang et al. showed that aberrant leukocyte infiltration and inflammation in the placenta were due to bile acid-induced activation of the GPBAR1/NF-*κ*B pathway [57, 58]. GPBAR1 can encode a membrane *G* protein-coupled receptor (TGR5), which plays a key role in bile acid homeostasis and liver lipids [59]. UDCA can compete with other bile acids for binding with GPBAR1 and thus inhibits bile acid-induced inflammatory response in trophoblasts and improves fetal survival in pregnant rats with obstructive cholestasis. Notably, inhibition of NF-*κ*B by andrographolide is more effective than UDCA in benefiting placentas and fetuses, suggesting a potentially better drug target for ICP management.

## 6. The Role of Bile Acid and Its Receptors

**Figure 4:**
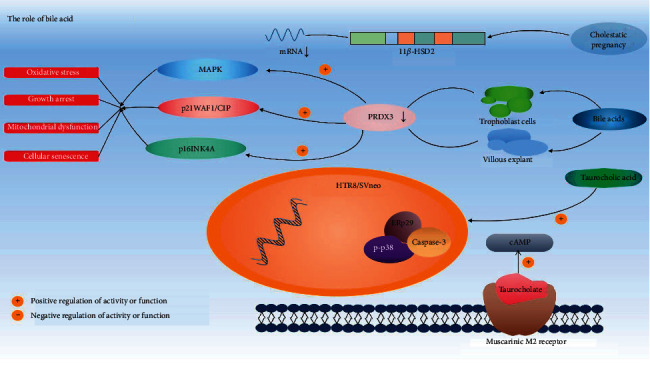
The role of bile acid in the pathogenesis of ICP. Taurocholate can bind to M2 receptors to inhibit cAMP. Taurocholic acid can upregulate ERp29, p-p38, and caspase-3 activities of HTR8/SVneo cells. The level of PRDX3 in trophoblast cells and villous explants treated with bile acids in vitro is downregulated, thereby inducing MAPK, p21WAF1/CIP, and p16INK4A, triggering oxidative stress, mitochondrial dysfunction, growth arrest, and cell senescence; The expression of 11*β*-HSD2 gene was reduced in cholestatic pregnancy. Adjustment events are displayed with arrows. The combination of wavy lines represents the DNA molecules in HTR8/SVneo cells, and a long rectangle shows the 11*β*-HSD2 gene fragment.

The disturbed bile acid homeostasis plays a key role in the pathogenesis of ICP (Figure 4). Research studies from the last 10 years illuminate how increased bile acids cause ICP from physiological, cellular, and molecular levels.

FXR, also known as a bile acid receptor, was found to be expressed in the placenta at a low level. Bile acids activate FXR by upregulating the expression of small heterodimer chaperones (SHP), resulting in the downregulation of the expression of transporters NTCP and OATP1 B1/3 involved in the uptake of bile acids [60]. FXR agonist could decrease bile acid levels and protect against the impairment of placentas induced by E2, probably through increasing the expressions of BSEP and antioxidant enzymes (PRDX1 and PRDX3) [61]. Zou et al. also found that CDCA can lower the levels of serum total bile acid by upregulating the expression of FXR and BSEP and then increasing the transport of the bile acids. However, raised hepatic bile acid concentrations during pregnancy in mice are associated with reduced FXR function. Milona et al. showed that both ablation and activation of FXR prevent the accumulation of hepatic bile acids during pregnancy, suggesting that the function of FXR may be perturbed during gestation [62]. They also found that serum from pregnant mice and humans can repress expression of the FXR target gene (small heterodimer partner, SHP) in liver-derived Fao cells, while ER antagonist abolished the repressive effects on SHP expression.

PPAR-*γ* can provide anti-inflammatory and protective effects in ICP through the NF-*κ*B pathway, participating in the occurrence and development of ICP. Peroxisome proliferator-activated receptor-*γ* (PPAR-*γ*) is an important subtype of the PPAR family. The activation of PPAR-*γ* can improve endothelial function, trigger antioxidant activity, and inhibit stress response [10]. NF-*κ*B is a nuclear transcription factor that can bind to DNA elements and regulate the expression of a variety of cytokines. It plays an important role in the immune response of Th1/Th2 cytokines [11], and the high expression levels of placental tissue NF-*κ*B play an important role in the pathogenesis of ICP. PPAR can promote the expression of antioxidant enzymes, thereby reducing the concentration of reactive oxygen species (ROS) and indirectly acting on NF-*κ*B [12]. Yan Zhang et al. collected and detected the levels of cytokines and bile acids in the serum of pregnant women. PPAR-*γ* and NF-*κ*B are highly expressed in placenta and HTR-8/SVneo cells, and serum cytokine levels are abnormal, suggesting that PPAR-*γ* and NF-*κ*B may play an important role in the pathogenesis of ICP [63]. In further research, it was found that PPAR-*γ* can inhibit the transcriptional activity of NF-*κ*B, tumor necrosis factor-*α* (TNF-*α*), interferon-*γ* (IFN-*γ*), and interleukin cytokines IL-1b and IL-2 [58]. Primary biliary cholangitis causes patients to have progressive duct reduction and cholestasis. For patients with the insufficient response or ursodeoxycholic acid intolerance, the FXR (NR1H4) agonist obeticholic acid can be used, and the pan-PPAR agonist bezafibrate can also serve as an alternative therapy [64].

Bile acids can also affect heart function. Taurocholate can bind to the muscarinic M2 receptor and serves as a partial agonist of this receptor in terms of the inhibitory effect on intracellular cAMP and negative chronotropic response [65], suggesting a new mechanism of bile acid-induced arrhythmia. UDCA can prevent ventricular conduction slowing and arrhythmia by restoring T-type calcium current in fetuses during cholestasis [66]. Moreover, Schultz et al. showed that UDCA can reduce fibroblast differentiation into myofibroblasts and activate potassium channels to induce hyperpolarisation of myofibroblasts [67], suggesting its application as an antifibrotic and antiarrhythmic drug for the treatment of failing hearts and fetal arrhythmia.

Neonates of ABCB11-deficient mothers have elevated pulmonary bile acids and altered pulmonary surfactant structure. Using animal models, Zhang et al. showed that maternal bile acid transporter deficiency in ATP-binding cassette, subfamily B member 11 (ABCB11) knockout mice promoted neonatal demise within 24 h of birth due to atelectasis-producing pulmonary hypoxia [68]. Moreover, through recording spontaneous periodic respiratory-related rhythmical discharge activity from hypoglossal nerves in the in vitro brainstem medulla slice of neonatal rats, Zhao et al. showed that bile acids could decrease respiratory cycle, inspiratory time, expiratory time, and integral amplitude [69].

How increased level of bile acids causes cell senescence and apoptosis is uncovered based on in vitro cell culture data. Zhang et al. showed that high concentration of taurocholic acid increased ERp29 expression, p-p38, and caspase-3 activity and the apoptotic index in trophoblast cell line HTR8/SVneo [70]. Wu et al. demonstrated that the level of peroxiredoxin 3 (PRDX3), a member of antioxidant enzymes family [71, 72], was downregulated in ICP placentas as well as bile acids-treated trophoblast cells and villous explant in vitro [73]. PRDX3 knockdown induced oxidative stress and mitochondrial dysfunction, growth arrest, and cellular senescence via activation of p38-mitogen-activated protein kinase (MAPK) and induction of p21WAF1/CIP and p16INK4A. Martineau et al. showed that the mRNA expression of placental 11*β*-hydroxysteroid dehydrogenase type 2 (11*β*-HSD2), which has protective role in preventing fetal exposure to excessive maternal cortisol, was reduced in human and murine cholestatic pregnancy [74].

Moreover, the molecular mechanism of how UDCA and other drugs for ICP functions is illuminated. CDCA and UDCA significantly induced reduction in AMP-activated protein kinase (AMPK) phosphorylation in sandwich cultured rat primary hepatocytes through activating cyclic AMP-dependent protein kinase (PKA) [75]. Emma et al. found that UDCA could protect the fetus of ICP patients by inhibiting OATP4A1-mediated bile acid transfer and TC-induced placental vasoconstriction [76]. However, in some trials on pregnant women with intrahepatic cholestasis, it was found that ursodeoxycholic acid could not effectively reduce the adverse perinatal outcome and had no clinically significant effect on maternal pruritus [77]. Yin Huang Mixture (YHHJ), which is herbal medicine, has a protective effect for E2-induced cholestasis and probably regulates hepatobiliary transporters, MRP2 and BSEP [78].

### 6.1. The Role of Matrix Metalloproteinase, Organelles, and Other Potential Players

Matrix metalloproteinases (MMPs) are involved in many illnesses affecting pregnant women. Some independent studies showed that both MMP-2 and MMP-9 were upregulated in ICP rats, which could be inhibited by epigallocatechin gallate (EGCG) or resveratrol treatment [79, 80]. Guo et al. confirmed that celastrol reduces intrahepatic cholestasis of pregnancy by inhibiting MMP-2 and MMP-9 [81]. However, UDCA could not inhibit the elevation of both MMPs. Both EGCG and resveratrol could ameliorate ICP symptoms, and resveratrol even displayed a better outcome than UDCA, suggesting potential novel drugs for ICP.

The dysfunction of the endoplasmic reticulum (ER) and mitochondria is implicated in the pathogenesis of ICP. Yu et al. reported obvious expansion of ER and increased expression of 78 kDa glucose-regulated protein (GRP-78) in trophocytes of ICP patient placental tissues [82]. Chao et al. studied the mTOR signal transduction pathway, which leads to endoplasmic reticulum stress, reducing the vitality of vegetative cells [83]. Mella et al. measured mitochondrial gene expression profiles and found dysregulation of NADH-ubiquinone oxidoreductase chain 4 L (MT-ND4 L) and several other genes [84]. Zou et al. found that endoplasmic reticulum protein 29 (ERp29) was upregulated in trophoblast cells (HTR-8/SVeno) of ICP patients [85].

Through placental gene-expression profiles and proteomics analysis, more genes and signaling pathways are found to be regulated in ICP. Wei et al. reported 392 differentially expressed genes between ICP and normal placental tissues [86]. Among these differentially expressed genes, 280 were upregulated and 112 were downregulated. These differentially expressed genes involved 20 categories including genes involved in transportation, cell growth, apoptosis, and immune response. Du performed similar analysis and found that the core regulatory genes were mainly involved in immune response, VEGF signaling pathway, and G-protein-coupled receptor signaling [87].

Comparative proteomics analysis found less differentially expressed proteins. He et al. reported 37 differentially expressed proteins, which involved in cytoskeleton activity, blood coagulation, and platelet activation as well as chaperones, heat shock proteins, RNA-binding and calcium-binding proteins, and various enzymes [88]. Zhang et al. found 38 differentially expressed proteins, among which 29 were upregulated and 9 were downregulated in ICP placenta [89]. The expression levels of ERp29, PRDX6, and MPO were further confirmed by Western blot. Koroglu et al. found that the levels of novel hepatokine fetuin B increased in patients with ICP [90]. Bioinformatics analysis suggested the involvement of apoptosis, oxidative stress, and lipid metabolism in the pathogenesis of ICP.

## 7. Conclusion and Perspective

ICP, although rare, is a serious health burden for both pregnant women and fetuses. Due to the lack of experimental research data related to its pathogenesis, treatment, and adverse outcomes, ICP is still a difficult-to-manage disease. According to our review, ICP is mainly caused by genetic, hormonal, environmental, and immunological factors. Immunological modifications are primary to cholestasis because intrahepatic cholestasis of pregnancy is an inflammatory disorder. Imbalance of immune function may destroy the immune microenvironment of pregnant women, leading to various pathological pregnancy reactions. Research pointed out that Th1-type cytokine TNF-*α* expression can be detected in the placenta tissue of normal pregnant women, and with the end of pregnancy, the TNF-*α* expression level gradually decreases and returns to the normal level [91]. In addition, IL-6 and IL-10 may also be involved in the pathophysiological process of ICP [92, 93].

UDCA, which has been used to treat ICP for a long time, actually has a minor beneficial effect for fetuses. The choleretic effects of UDCA are potentially mediated by its activity at the MRP2 or MRP3 receptors. Another drug rifampin, which can inhibit bile acid reuptake by hepatocytes and enhance bile acid detoxification, potentially strengthens the efficacy of UDCA. Also, investigations of guar gum and S-adenosyl-L-methionine have demonstrated limited success in the treatment of ICP [4]. Thus, more effective drugs need to be studied. This article summarizes several signal pathways from hormone levels, bile acids, genetic regulators, inflammatory factors, hypoxia, and other possible ways, further revealing some of the mechanisms of ICP. The advancement in our understanding of the molecular pathogenesis of ICP has uncovered more potential drug targets, whose clinical application needs to be further explored. In short, we have more choices to handle ICP than 10 years ago. New drugs are hoped to be developed.

## Data Availability

The data used to support the findings of this study are available from the corresponding author upon request.
